# Clinical assessment of small bowel capsule endoscopy in pediatric patients

**DOI:** 10.3389/fmed.2024.1455894

**Published:** 2024-10-16

**Authors:** Lin Li, Xue Zhan, Jun Li, Shuyuan Li, Yuxia Chen, Liyan Yang, Yuting Wang

**Affiliations:** Department of Gastroenterology, Ministry of Education Key Laboratory of Child Development and Disorders, China International Science and Technology Cooperation Base of Child Development and Critical Disorders, Chongqing Key Laboratory of Pediatrics, National Clinical Research Center for Child Health and Disorders, Children’s Hospital of Chongqing Medical University, Chongqing, China

**Keywords:** capsule endoscopy, diagnosis, small intestine, children, abdominal pain (MeSH)

## Abstract

**Introduction:**

Small bowel capsule endoscopy is a first-line examination method for small bowel diseases, which can find small intestinal lumen and mucosal lesions.

**Methods:**

We retrospectively assessed patients who underwent small bowel capsule endoscopy between September 2020 and May 2023 to examine their clinical and small bowel capsule endoscopic data, aiming to provide insights into the application of this technique in pediatric patients with small intestinal diseases.

**Results:**

All instances of capsule retention were successfully resolved through enteroscopy. Of the 1140 children who completed the capsule endoscopy, 97.46% (1111/1140) underwent a comprehensive examination of the entire small intestine without experiencing any discomfort. Capsule endoscopy yielded abnormal findings in 672 cases, with a positive detection rate of 58.95%. Among the positive results, intestinal mucosal inflammatory lesions were the most prevalent, occurring in 292 cases (43.45%), followed by ulcerative or erosive lesions in 236 cases (35.12%), diverticulum in 54 cases (8.04%), and vascular lesions in 30 cases (4.46%). Lymphangiectasis was observed in 16 cases (2.38%). The distribution of positive lesions did not exhibit significant gender-based differences, but there were variations among different age groups. Among all children who completed the small bowel capsule endoscopy, the most frequently reported symptom was abdominal pain (815/1140 cases, 71.49%), followed by 130 cases (11.40%) of bloody stools or melena.

**Discussion:**

Small bowel capsule endoscopy is well-tolerated and safe in children, carrying significant clinical importance for diagnosing abdominal pain and obscure gastrointestinal bleeding in pediatric patients.

## 1 Introduction

Small bowel capsule endoscopy (SBCE) is a non-invasive technique that utilizes optical principles to capture images of intestinal lesions, enabling direct observation of the intestinal mucosa and providing diagnostic imaging of the small intestine. SBCE received approval from the United States Food and Drug Administration for evaluating small intestinal diseases in adults in 2001, and subsequently, in 2009, it was also approved for use in children aged 2 years or older.

Over the past two decades, SBCE has become the cornerstone of small bowel diagnostics, offering a minimally invasive alternative to traditional endoscopic methods. Its widespread adoption in adult medicine has been well-documented, leading to significant advancements in the diagnosis and management of small bowel conditions such as Crohn’s disease, obscure gastrointestinal bleeding, and small bowel tumors. However, despite its established efficacy in adults, the application of SBCE in pediatric populations remains less explored.

Children present unique challenges in gastrointestinal diagnostics due to differences in anatomy, physiology, and the spectrum of diseases compared to adults. Additionally, the technical and procedural aspects of SBCE, such as capsule ingestion and transit times, may vary significantly between children and adults, potentially influencing diagnostic outcomes. While some studies have begun to address these differences, there is still a notable gap in the literature regarding the use of SBCE in pediatric patients, particularly in understanding its diagnostic yield, safety profile, and clinical impact across different age groups.

This study aims to bridge this gap by providing comprehensive data on the use of SBCE in children, evaluating its efficacy in diagnosing small bowel diseases, and exploring the factors that may influence its diagnostic accuracy in this population. Through this research, we hope to contribute valuable insights into the optimization of SBCE protocols for pediatric patients and enhance the overall management of small bowel diseases in children.

## 2 Patients and methods

### 2.1 Patients

We conducted a retrospective study on pediatric patients who were under 18 years old referred for small bowel capsule endoscopy (SBCE) at the Affiliated Children’s Hospital of Chongqing Medical University from September 2020 to May 2023. The clinical data retrieved included gender, age, presenting symptoms, examination details and clinical diagnoses.

Our study followed the indications outlined in Chinese guidelines for SBCE, the guidelines for wireless capsule endoscopy in children and adolescents from Spain, and clinical practice guidelines for capsule endoscopy by the American Society of Gastroenterology (1–3).

Therefore, our inclusion criteria consist of children undergoing capsule endoscopy for suspected small bowel disease, which encompassed small intestinal bleeding, unexplained iron deficiency anemia, Crohn’s disease, small intestinal neoplasms, polyposis, celiac disease, and NSAIDs-associated small intestinal mucosal lesions. The exclusion criteria include children who are unable to complete capsule endoscopy for any reason, as well as those with cardiac pacemakers or other implanted electronic devices.

Approval was obtained from the Ethics Committee of the Children’s Hospital of Chongqing Medical University, and informed consent forms were signed by the parents or legal guardians of all participating children who underwent SBCE.

### 2.2 Process

We used the PillCam^TM^ SB3 capsule endoscope system for our examinations. Patients adhered to a preparation regimen that involved a 24-h transition to a low-residue and semi-liquid diet, followed by a 3-h fasting period immediately before the procedure. To clear the intestines, the patients were given a compound polyethylene glycol electrolyte solution (25 mL/kg) at both 12 h and 3 h prior to the procedure, with each intake completed within 1 h. Three hours before the procedure, each patient was also instructed to ingest 30 mL of silicone oil. Following capsule ingestion, the patients were kept in a right supine position with a 30-degree upper body elevation. In cases where the capsule failed to enter the duodenum within 2 h post-ingestion, a domperidone tablet (0.3 mg/kg, max 10 mg) was administered. If duodenal entry did not occur after 4 h or if the patient faced swallowing difficulties, gastroscopy assistance was employed to guide the capsule to the distal duodenum. Normal diet resumption occurred upon capsule entry into the colon or examination completion.

Our primary observation endpoint was that SBCE passed through the ileocecal valve or the capsule’s battery was exhausted before passing through the ileocecal valve, and the secondary observation endpoint was the capsule’s excretion from the body. The data collected included the transit time of the capsule through the small intestine, capsule retention rate, and small intestinal pathological findings.

### 2.3 Statistical analysis

All statistical analyses were performed using the SPSS v26 software. Metrological data are expressed using mean ± standard deviation (x ± s), while classified data are expressed using number of cases and their proportion (*n*, %). According to different ages, the children were divided into three groups: ≤5 years old, 6–11 years old and ≥12 years old (4). *T*-test was used to compare two means, and the chi-square test was used for multiple groups. *p* < 0.05 was used to determine statistical significance.

## 3 Results

### 3.1 Patient characteristics

In this study, a total of 1,143 children were initially included, among whom 1,140 successfully completed SBCE, resulting in a completion rate of 99.74% (1,140/1,143). The average age of the 1,140 children was 10.5 ± 3.1 years, comprising 676 males (59.30%) and 464 females (40.70%).

Among these 1,140 children, 1,111 achieved SBCE passage into the colon through the ileocecal valve, thereby completing the entire small bowel examination and representing a completion rate of 97.46%. No instances of discomfort, such as abdominal pain or vomiting, were reported during the small intestine examination. In 29 cases, when the battery of SBCE was exhausted, the capsule endoscope was still in the small intestine and did not enter the colon through the ileocecal valve.

Among the 1,140 children who underwent SBCE, 672 cases (58.9%) exhibited abnormalities detected by SBCE, indicating a substantial positive detection rate (see Table 1).

**Table 1:** Baseline characteristics of the 1,140 investigated patients.

Baseline information	Data
Age (year)	10.5 ± 3.1
Male	676
Female	464
Swallowed orally	864
Gastroscope assistance	276^a^
Complete examination of the small intestine (%)	1,111 (97.46)
Capsule retention (%)	2 (0.18)
Abnormalities (%)	672 (58.95)

a Two SBCE were retained in the stomach and were finally released to the distal descending part of the duodenum by gastroscopy.

### 3.2 Capsule retention

We defined the capsule retention as the capsule was not naturally excreted within 14 days. Among the 1,140 children, 2 had capsule retention, showing a retention rate of 0.18%, with the retention site being the small intestine. One of the patients with capsule retention had symptoms of abdominal pain, and the other had no symptoms. The retention capsules were subsequently removed via enteroscopy. Of note, both of them were diagnosed with Crohn’s disease.

### 3.3 Capsule intake mode

Among the 1,140 children who successfully completed the examination, 864 cases (75.79%) swallowed the capsules orally, while 274 cases (24.03%) had the capsules directed to the distal end of the descending duodenum via gastroscopy. Additionally, 2 cases (0.18%) initially ingested the capsules orally but experienced retention in the stomach, eventually requiring gastroscopic intervention to facilitate passage to the distal part of the descending duodenum. The children were categorized into three age groups: ≤5 years old, 6–11 years old, and ≥12 years old. In the ≤5-year-old group, which consisted of 92 children, 27 cases involved oral capsule ingestion, while 65 cases required assistance through gastroscopy. Among the 647 children aged 6–11 years, 499 cases opted for oral capsule ingestion, and 148 cases underwent gastroscopic assistance. In the ≥12-year-old group comprising 401 cases, 338 children swallowed the capsules orally, and 63 received assistance via gastroscopy. Statistical analysis using the chi-square test indicated a significant difference among the three age groups: *χ*^2^ = 124.6 > 5.99 (*p* = 0.05, *ν* = 2) and *p* < 0.05, indicating a notably higher proportion of gastroscopy-assisted capsule delivery in the age group of children ≤5 years old (see Table 2).

**Table 2:** Comparison of SBCE intake mode in children of different age groups.

Age	Cases	Orally	Gastroscope assistance	Gastroscope ratio
≤5 years	92	27	65	70.65
6–11 years	647	499	148	22.87
≥12 years	401	338	63	15.71
Total	1,140	864	275	24.12

*χ*^2^ = 124.6 > 5.99 (*p* = 0.05, *ν* = 2), *p* < 0.05.

### 3.4 Gastric and small bowel transit time

1) Gastric transit time (GTT): We calculated GTT for a total of 845 cases among children who orally ingested SBCE and recorded GTT data. Those who did not have GTT records were excluded. Finally, a total of 845 cases were included in the calculation, giving a GTT of 68.1 ± 78.6 min (0.5, 837).
2) Small bowel transit time (SBTT): SBTT was determined for 1,106 cases, excluding those in which the capsules did not pass through the ileocecal valve (i.e., due to electricity of the capsule being exhausted) or lacked recorded SBTT data. The average SBTT was 254.9 ± 109.1 min (40, 739).
3) The influence of gender on SBTT: Among 453 female children, the SBTT averaged 261.1 ± 107.2 min (60, 669), while in 653 male children, it averaged 250.6 ± 110.3 min (40, 739). A *Z*-test comparing the two groups resulted in a *Z*-score of 1.58, which was below the critical value of 12.7 (*p* = 0.05, *ν* = 1), indicating no significant difference in SBTT between genders (*p* > 0.05).
4) The influence of age on SBTT: SBTT varied across age groups, measuring 292.3 ± 144.9 min (63, 667) in the ≤5 years old group, 241.8 ± 100.2 min (48, 739) in the 6–11 years old group, and 267.7 ± 110.2 min (40, 733) in the ≥12 years old group. *Z*-tests between two of the three groups revealed a significant difference between the 6–11 years old group and the other two (*p* < 0.05), indicating that the SBTT is shortest in the 6–11 years old group. However, no significant difference was observed between the ≤5 years old and ≥12 years old groups (*p* > 0.05). These findings suggest age-related variations in SBTT, with the shortest transit time observed in the 6–11 years old group, while no significant difference exists between the ≤5 years old and ≥12 years old groups (Table 3).
5) The influence of SBCE findings on SBTT was assessed by categorizing the children into three groups based on their SBCE results: the SBCE negative group, the enteritis group, and the ulcer or erosion group. Exclusion criteria included children without recorded SBTT data and those who did not pass the ileocecum. The SBTT for the negative group was 235.6 ± 96.7 min (40, 708), the enteritis group recorded an SBTT of 246.3 ± 100.4 min (48, 718), and the ulcer erosion group exhibited an SBTT of 311.3 ± 151.0 min (66, 853). *Z*-tests were conducted between two of the three groups, revealing a significant difference in the ulcer or erosion group compared to both the negative group and the enteritis group (*p* < 0.05). However, no significant difference was observed between the enteritis group and the negative group (*p* > 0.05). These results indicate that the SBTT was longer in the ulcer or erosion group compared to the negative and enteritis groups, while no significant difference was observed between the enteritis and negative groups (Table 3).

**Table 3:** Comparison of SBTT in children of different genders and ages and major lesions.

Variables	Gender		Age			Lesions		
	Male	Female	≤5 years	6–11 years	≥12 years	Negative group	Enteritis group	Ulcer or erosion group
No. of cases	653	453	90	633	383	463	292	231
SBTT (minutes)	250.6 ± 110.3	261.1 ± 107.2	292.3 ± 144.9	241.8 ± 100.2	267.7 ± 110.2	235.6 ± 96.7	246.3 ± 100.4	311.3 ± 151.0

Gender: *z* = 1.58 < 12.7 (*p* = 0.05, *ν* = 1), *p* > 0.05.

### 3.5 SBCE findings

Among the 1,140 cases examined by SBCE, 672 cases (58.95%) had positive findings in the small intestine. These findings included intestinal mucosal inflammatory lesions, which were identified in 292 cases (43.45%), followed by ulcers or erosion found in 236 cases (35.12%). Additionally, diverticulum was detected in 54 cases (8.04%), vascular lesions in 30 cases (4.46%), and lymphangiectasis in 16 cases (2.38%) (Table 4).

**Table 4:** Analysis of SBCE findings in 1,140 cases.

Diagnosis	Cases
Negative	468
Intestinal mucosal inflammatory lesion	292
Ulcer/erosion	236
Diverticulum	54
Vascular lesion	30
Lymphatic dilatation	16
Polyp	22
Parasite	4
Others	18

A total of 468 cases were categorized as negative in SBCE (the negative group), comprising 264 males and 204 females. SBCE revealed 292 cases of intestinal mucosal inflammatory lesions (the enteritis group), with 170 males and 122 females, and 236 cases of ulcers or erosions (the ulcer or erosion group), including 151 males and 85 females. Additionally, there were 54 cases of diverticulum-associated intestinal mucosal inflammatory lesions (the diverticulum group), consisting of 40 males and 14 females, 30 cases of small intestinal vascular lesions (the vascular disease group) with 16 males and 14 females, and 16 cases of small intestinal lymphangiectasis (the lymphangiectasis group), including 9 males and 7 females. Chi-square test conducted among these groups yielded a *χ*^2^ value of 9.29, which is less than the critical value of 11.07 (*p* = 0.05, *ν* = 5), indicating no significant difference among the various SBCE findings (*p* > 0.05).

In the negative group, there were 230 cases in the ≤5 years old category, 308 cases in the 6–11 years old group, and 130 cases in the ≥12 years old group. In the enteritis group, there were 18 cases in the ≤5 years old category, 165 cases in the 6–11 years old group, and 109 cases in the ≥12 years old group. For the ulcer or erosion group, 23 cases were in the ≤5 years old category, 110 cases in the 6–11 years old group, and 103 cases in the ≥12 years old group. In the diverticulum group, 7 cases were in the ≤5 years old category, 24 cases in the 6–11 years old group, and 23 cases in the ≥12 years old group. The vascular disease group included 9 cases in the ≤5 years old category, 7 cases in the 6–11 years old group, and 14 cases in the ≥12 years old group. Finally, the lymphangiectasis group consisted of 1 case in the ≤5 years old category, 9 cases in the 6–11 years old group, and 6 cases in the ≥12 years old group. A chi-square test conducted among these different groups resulted in a *χ*^2^ value of 56.3, which is greater than the critical value of 18.307 (*p* = 0.05, *ν* = 10), indicating statistical differences in SBCE findings across different age groups (Table 5).

**Table 5:** Comparison of gender and age of children in different SBCE findings groups.

Lesions	Gender		Age		
	Male	Female	≤5 years	6–11 years	≥12 years
Negative	264	204	30	308	130
Intestinal mucosal inflammatory lesion	170	122	18	165	109
Ulcer/erosion	151	85	23	110	103
Diverticulum	40	14	7	24	23
Vascular lesion	16	14	9	7	14
Lymphatic dilatation	9	7	1	9	6

Gender group: *χ*^2^ = 9.29 < 11.07 (*p* = 0.05, *ν* = 5), *p* > 0.05; age group: *χ*^2^ = 56.3 > 18.307 (*p* = 0.05, *ν* = 10), *p* < 0.05.

Among the 672 cases with positive lesions, 58 children were <5 years old and most presented with an ulcer or erosion (23 cases, 39.66%). In the 6–11 age group, which consisted of 315 children, the most prevalent findings were intestinal mucosal inflammatory lesions (165 cases, 52.38%). In the ≥12 years old group, comprising 255 children, the predominant findings were also intestinal mucosal inflammatory lesions (109 cases, 42.75%).


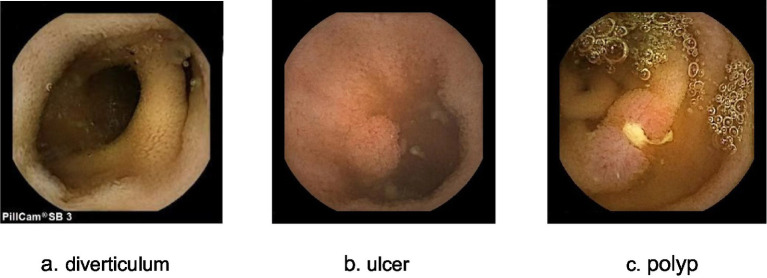


### 3.6 Analysis of the chief complaint

Of the 1,140 children who successfully completed the examination, 816 presented with abdominal pain as their chief complaint, making it the most frequent reason for undergoing SBCE examination. In addition, 130 cases reported symptoms of bloody stool or melena, while 53 cases were subjected to SBCE as part of a Crohn’s disease follow-up plan. Furthermore, 29 cases presented with diarrhea, 18 cases reported unexplained anemia, 20 cases experienced vomiting, and 75 cases exhibited symptoms such as fever, rash, nausea or other medical conditions (Table 6).

**Table 6:** Comparison of sex and age of children with different chief complaints.

Complaints	Total	Proportion (%)	Gender		Age		
			Male	Female	≤5 years	6–11 years	≥12 years
Abdominal pain	815	71.49	477	338	45	514	256
Bloody stool or melena	130	11.4	82	48	23	59	48
Crohn’s disease follow-up	53	4.65	34	19	1	14	38
Diarrhea	29	2.54	21	8	5	9	15
Unexplained anemia	18	1.59	10	8	2	8	8
Vomiting	20	1.75	11	9	0	11	9
Others	75	6.58	41	34	15	33	27

Gender: *χ*^2^ = 4.36 < 12.59 (*p* = 0.05, *ν* = 6), *p* > 0.05; age: *χ*^2^ = 92.5 > 21 (*p* = 0.05, *ν* = 12), *p* < 0.05.

Regarding gender distribution among different chief complaint groups, there were 815 patients in the abdominal pain group, including 477 males and 338 females, 130 cases in the blood stool or melena group, comprising 82 males and 48 females, and 53 cases in the Crohn’s disease follow-up group, with 34 males and 19 females. Chi-square test conducted among these groups resulted in a *χ*^2^ value of 4.36, which is less than the critical value of 12.59 (*p* = 0.05, *ν* = 6), indicating no statistical difference in gender distribution among the various chief complaint groups (*p* > 0.05).

Age subgroup analysis revealed variations among the different complaint groups. In the abdominal pain group, there were 45 children aged ≤5 years old, 514 children aged 6–11 years, and 256 children aged ≥12 years old. In the blood stool or melena group, 23 children were ≤5 years old, 59 children were aged 6–11 years, and 48 children were ≥12 years old. In the Crohn’s disease follow-up group, 1 child was ≤5 years old, 14 children were aged 6–11 years, and 38 children were ≥12 years old. A chi-square test conducted among these different groups resulted in a *χ*^2^ value of 92.5, which exceeds the critical value of 21 (*p* ≤ 0.05, *ν* = 12), indicating significant differences in age distribution among the various complaint groups (Table 6).

In the abdominal pain group, which included 815 cases, SBCE results showed no evident abnormalities in 390 cases. However, there were 225 cases of intestinal mucosal inflammatory lesions, 138 cases of ulceration or erosion, 12 cases of intestinal diverticulosis, 12 cases of vascular lesions, 14 cases of lymphangiectasia, 8 cases of polyps, 4 cases of parasitic lesions, and 11 cases of other lesions. In the blood stool or melena group, comprising 130 cases, SBCE revealed no apparent abnormalities in 33 cases. However, there were 19 cases of intestinal mucosal inflammatory lesions, 25 cases of ulceration or erosion, 35 cases of intestinal diverticulosis, 12 cases of vascular lesions, 1 case of lymphangiectasia, 3 cases of polyps, and 2 cases of other lesions (Table 7).

**Table 7:** Analysis of main complaint of SBCE findings.

Lesions									
Complaint	Negative	Intestinal mucosal inflammatory lesion	Ulcer/erosion	Diverticulum	Vascular lesion	Lymphatic dilatation	Polyp	Parasite	Others
Abdominal pain	390	225	138	12	12	14	8	4	12
Blood stool/melena group	33	19	25	35	12	1	3	0	2

### 3.7 Consistency between SBCE findings and final clinical diagnosis

Among the 1,140 SBCE cases, 288 received a final diagnosis of enteritis, among whom 212 cases showed SBCE findings consistent with the final diagnosis, while 71 cases had negative SBCE results. In addition, 248 cases were diagnosed with gastritis, among them, 38 cases were diagnosed with gastritis based on gastric mucosal lesions detected by SBCE. Additionally, SBCE did not reveal any significant lesions in 205 cases. We consider a negative SBCE finding as an important criterion for the diagnosis of gastritis, therefore, these 205 cases are still deemed to match the final diagnosis. Thus, a total of 243 cases had SBCE results consistent with the final diagnosis. Furthermore, 115 cases were diagnosed with ulceration or erosion of the digestive tract. Among them, 90 cases had SBCE findings that corresponded to the final diagnosis, while 23 cases had negative SBCE results (Table 8). Overall, 653 cases demonstrated SBCE findings concordant with the final diagnosis, resulting in an overall diagnostic agreement rate of 83.16%.

**Table 8:** Classification of final diagnosis of 1,140 children.

Diagnosis	Total	Consistent	Inconsistent	Negative of SBCE
Enteritis	288	212	76	71
Gastritis	248	243	5	205
Ulcer or erosion of the digestive tract	115	90	25	23
Crohn’s disease	111	106	5	5
Functional gastrointestinal disease	107	107	0	90
Vasculitis or purpura	79	70	9	7
Inflammatory bowel disease (unclassified)	58	40	18	16
Gastrointestinal polyposis	34	21	13	13
Meckel’s diverticulum	28	25	3	2
Others	72	34	38	35
Total	1,140	948	192	467

In the group of children with intestinal mucosal inflammatory disease, 285 cases (168 males and 117 females) were consistent with the final diagnosis, and the resulting coincidence rate was 88.4% (285/292). In the ulcer erosion group, 233 cases (150 males and 83 females) matched the final diagnosis, yielding a high coincidence rate of 98.7% (233/236). Furthermore, in the diverticulum group, the findings in 52 children (39 males and 13 females) were consistent with the final diagnosis, resulting in a coincidence rate of 96.3% (52/54). Chi-square test conducted across multiple groups showed a *χ*^2^ value of 7.5, which is less than the critical value of 14 (*p* < 0.05, *ν* = 7), indicating that gender did not significantly influence the coincidence of SBCE findings with the final clinical diagnosis (Table 9).

**Table 9:** Comparison of gender and age distribution of children with positive lesions in accordance with clinical diagnosis.

Lesions	Total coincidence cases	Gender		Age		
		Male	Female	≤5 years	6–12 years	≥12 years
Intestinal mucosal inflammatory lesion	285	168	117	18	162	105
Ulcer/erosion	233	150	83	23	107	103
Diverticulum	52	39	13	6	23	23
Vascular lesion	30	16	14	9	7	14
Lymphatic dilatation	14	8	6	1	7	6
Polyp	22	12	10	2	12	8
Parasite	3	2	1	0	2	1
Others	8	4	4	1	4	3

Gender: *χ*^2^ = 7.5 < 14 (*p* = 0.05, *ν* = 7), *p* > 0.05; age: *χ*^2^ = 28.8 > 23.7 (*p* = 0.05, *ν* = 14), *p* < 0.05.

Age subgroup analysis (Table 9) showed that in the enteritis group, there were 8 cases in the ≤5 years old category, 66 cases in the 6–11 years old category, and 38 cases in the ≥12 years old category. In the ulcer erosion group, there were 10 cases in the ≤5 years old category, 52 cases in the 6–11 years old category, and 36 cases in the ≥12 years old category. In the diverticulum group, there were 3 cases in the ≤5 years old category, 12 cases in the 6–11 years old category, and 13 cases in the ≥12 years old category. Chi-square test conducted across multiple groups showed a *χ*^2^ value of 28.8, which exceeds the critical value of 23.7 (*p* = 0.05, *ν* = 14), indicating a significant difference in age distribution among the groups with positive lesions.

### 3.8 Analysis of the coincidence between the chief complaint and SBCE findings

Concerning the chief complaint, among the 815 children in the abdominal pain group, 411 cases were found to be concordant with the final diagnosis through capsule endoscopy, resulting in a coincidence rate of 50.4%. In contrast, among the 130 children in the bloody stool or melena group, 95 cases exhibited findings consistent with the final diagnosis via capsule endoscopy, yielding a coincidence rate of 73.1%.

Subgroup analysis: Among the children whose SBCE findings were consistent with the clinical diagnosis, there were 411 cases with abdominal pain, comprising 246 males and 165 females. Additionally, there were 95 children with bloody stool or melena, including 61 males and 34 females. A chi-square test was conducted to compare the two groups, resulting in a *χ*^2^ value of 0.6, which is less than the critical threshold of 3.84 (*p* = 0.05, *ν* = 1), suggesting that gender differences may not significantly impact the correct diagnosis rate between the two groups with different chief complaints (Table 10).

**Table 10:** Comparison of the consistency of main complaints with clinical diagnosis in children of different genders and ages.

Complaint	Gender		Age		
	Male	Female	≤5 years	6–11 years	≥12 years
Abdominal pain	246	165	27	236	148
Bloody stool or melena	61	34	16	39	40

Gender: *χ*^2^ = 0.6 < 3.84 (*p* = 0.05, *ν* = 1), *p* > 0.05; age: *χ*^2^ = 14.2 > 5.99 (*p* = 0.05, *ν* = 2), *p* < 0.05.

Subgroup analysis: Among the children whose SBCE findings were consistent with the clinical diagnosis, there were 411 cases with abdominal pain, consisting of 27 cases aged ≤5 years, 236 cases aged 6–11 years, and 148 cases aged ≥12 years. Additionally, there were 95 children with bloody stool or melena, including 16 cases aged ≤5 years, 39 cases aged 6–11 years, and 40 cases aged ≥12 years. Chi-square test conducted between the two groups showed a *χ*^2^ value of 14.2, which exceeds the critical value of 5.99 (*p* = 0.05, *ν* = 2), thereby indicating that age differences may indeed impact the correct diagnosis rate among children in the two groups with different chief complaints (Table 10).

## 4 Discussion

SBCE, which has become the primary diagnostic tool for small intestinal diseases, is a non-invasive technique that utilizes optical principles to directly visualize the mucous membranes of the small intestine, large intestine, and esophagus (5). It was approved for children over 2 years old in 2009 (6), and its reported minimum age in children is 8 months (7). In this study, the youngest patient included was 16 months old, who was referred for SBCE due to a history of “four episodes of bloody stool in 3 days.” SBCE revealed the presence of an ileal diverticulum, which was suspected to be Meckel’s diverticulum. However, abdominal ultrasound and technetium 99 m radionuclide scanning yielded normal results, and the family declined laparoscopic exploration; thus, a definitive diagnosis could not be established.

In this study, 1,111 out of the 1,140 patients successfully underwent a complete examination of the entire small intestine, resulting in a complete examination rate of 97.46%, comparable to the findings of a study by Iwama et al. (8), which reported a complete examination rate of 89.1% in 163 out of 183 patients. Notably, a study by Jensen et al. (9) that examined 117 children revealed that the use of gastroscopy-assisted placement of SBCE was associated with a higher risk of incomplete examination of the entire small intestine, possibly due to the effect of anesthetics, which led to reduced peristalsis of the small intestine during endoscopy and subsequent resting state, which were similar to our observations in this present study. Among the 29 children whose examination of the small intestine could not completed, 11 had ingested the capsules through gastroscopy, constituting 3.99% of the children who underwent gastroscopy-assisted capsule placement. Additionally, 18 cases involved oral ingestion of capsules, representing 2.08% of children with oral swallowing.

This study observed that children who completed the entire intestinal examination did not experience discomfort such as abdominal pain or vomiting during the procedure, indicating that capsule endoscopy is a safe and well-tolerated diagnostic tool in pediatric patients. The primary complication associated with SBCE examination was capsule retention, defined as the failure of the capsule to naturally exit the intestine within a two-week period. In our study, two children experienced capsule retention, resulting in a retention rate of 0.18%. Ultimately, both capsules were successfully removed through enteroscopy. These findings are consistent with a large-scale study involving 5,593 adult patients undergoing capsule endoscopy at a tertiary hospital, where only 0.3% of asymptomatic patients developed capsule retention (10), which is similar to our study. In our investigation, the two cases of capsule retention led to a diagnosis of Crohn’s disease with intestinal stenosis, which is in line with the findings reported by Atay et al. (11) A retrospective study conducted by Cohen (12) on 1,013 children who underwent SBCE revealed a capsule retention rate of 2.3%. While this retention rate was higher than our present study, it could be attributed to the elevated prevalence of Crohn’s disease in their study population (596 out of 1,013 cases). Both the findings from Cohen’s study and our own emphasize the increased risk of capsule retention in children with Crohn’s disease, underscoring the need for careful evaluation by healthcare providers before conducting SBCE examinations in these patients.

In our study, 274 children encountered challenges either due to difficulty swallowing the capsule or an unwillingness to do so. In these cases, the capsule was introduced to the distal end of the descending duodenum with the assistance of a gastroscope. Additionally, two children initially attempted to swallow the capsule orally but experienced retention in the stomach. Subsequently, the capsules were successfully ingested with the aid of a gastroscope. Upon analyzing the relationship between age and capsule intake, as anticipated, we found that younger children (≤5 years old) exhibited a greater reliance on gastroscopy-assisted capsule placement. This age group also demonstrated longer SBTT, consistent with the results reported by Burgess et al. (13), which may be related to the decrease in gastrointestinal motility caused by anesthetics.

In our study, we observed that the SBTT was shortest in children aged 6–11 years. We hypothesized that this finding may be due to following factors. For children aged ≤5 years, the reliance on gastroscopy-assisted capsule placement, which is more common in this age group, may have contributed to a prolonged SBTT. In contrast, for children aged ≥12 years, our previous findings suggest that SBTT is longer in those with ulcers or erosions compared to those with negative findings or enteritis. Since the prevalence of ulcers or erosions is higher in children aged ≥12 years than in those aged 6–11 years, this likely explains the longer average SBTT in the older age group.

In our study, we observed an average GTT of 68.1 ± 78.6 min and a SBTT of 254.9 ± 109.1 min. When examining the relationship between gender and SBTT, we found no significant gender-based differences, indicating that gender does not significantly influence SBTT. Additionally, our study aligns with the findings from Lasa’s et al. (14) research, which demonstrated a strong positive correlation between SBTT and pathological changes. Consistent with this, our study reveals a similar pattern: SBTT was notably prolonged in the ulcer or erosion group compared to the negative and enteritis groups. This prolonged SBTT may be attributed to factors like the rough texture of the intestinal mucosa, hyperemia, intestinal stenosis, and reduced peristalsis.

Our study revealed that the most common complaint among patients undergoing SBCE examination was abdominal pain, accounting for 815 cases (71.49%), followed by bloody stool or melena in 130 cases (11.40%). This pattern aligns with the findings from a study by Wu et al. (15) on the use of SBCE in children while also differing from the trends observed in adults, where obscure gastrointestinal bleeding serves as the primary indication for SBCE (16). The difference in chief complaints between children and adults undergoing capsule endoscopy may be attributed to differences in their underlying diseases. Furthermore, our analysis indicated that there were no significant gender-based differences in the distribution of complaints among different groups, while variations in age distribution were observed, suggesting the presence of distinct underlying diseases across different age groups among children.

Among the 1,140 children in this study, positive lesions were found in 672 cases, with an overall positive rate of 58.95%, which is similar to previous studies (8, 15). Among the positive lesions detected through SBCE, the most prevalent changes were inflammatory alterations of the intestinal mucosa, followed by ulcers or erosions, diverticula, vascular lesions, and lymphatic dilatation. It’s worth noting that our analysis showed no significant gender-based differences in the distribution of positive lesions, while the distribution was different in different age groups.

There are some limitations associated with this study. First, due to its retrospective nature, there might be certain biases in the reported findings. Second, we conducted a basic descriptive analysis and did not perform comprehensive follow-ups or investigations. Third, similar to other related investigations, we faced the challenge of lacking a gold standard to assess the accuracy of capsule endoscopy and relied on SBCE findings as a basic measure of disease assessment, diagnosis rates and positive outcomes. Therefore, determining the exact ratio of false positives to false negatives was difficult, underscoring the need for additional evaluations, such as enteroscopy or alternative diagnostic methods.

## 5 Conclusion

Small bowel capsule endoscopy (SBCE) proves to be a safe and well-tolerated diagnostic tool for children, offering significant clinical value in the evaluation of small bowel diseases. Our study demonstrates that SBCE exhibits a high diagnostic concordance with final diagnoses, effectively identifying conditions such as Crohn’s disease, abdominal pain, and gastrointestinal bleeding in the pediatric population. The high rate of positive detection underscores the efficacy of SBCE in accurately diagnosing small bowel disorders in children, thereby providing a reliable and minimally invasive alternative to traditional diagnostic methods. This emphasizes the importance of incorporating SBCE into clinical practice for the management of pediatric gastrointestinal conditions, ultimately enhancing patient care and diagnostic accuracy.

## Data Availability

The raw data supporting the conclusions of this article will be made available by the authors, without undue reservation.

## References

[1] National Clinical Research Center for Digestive Diseases (Shanghai), National Digestive Endoscopy Improvement System, Capsule Endoscopy Group of Chinese Society of Digestive Endoscopology, Capsule Endoscopy Collaborative Group of Digestive Endoscopy Branch of Shanghai Medical Association. Chinese guideline on small-bowel capsule gastroscopy (condensed edition, 2021, Shanghai). Chin J Dig. (2021) 41:509–13. doi: 10.3760/cma.i.cn311367-20210508-00265

[2] Enns RA Hookey L Armstrong D Bernstein CN Heitman SJ Teshima C. Clinical practice guidelines for the use of video capsule endoscopy. Gastroenterology. (2017) 152:497–514. doi: 10.1053/j.gastro.2016.12.032 28063287

[3] Argüelles-Arias F Donat E Fernández-Urien I Alberca F Argüelles-Martín F Martínez MJ. Guideline for wireless capsule endoscopy in children and adolescents: a consensus document by the SEGHNP (Spanish Society for Pediatric Gastroenterology, Hepatology, and Nutrition) and the SEPD (Spanish Society for Digestive Diseases). Rev Esp Enferm Dig. (2015) 107:714–31. doi: 10.17235/reed.2015.3921/2015, PMID: 26671584

[4] Fang JZ Ling SK Ying S. Zhu futang practice of pediatrics. 8th ed. Beijing: The People’s Health Press Co., Ltd. (2014).

[5] Rondonotti E Spada C Adler S May A Despott EJ Koulaouzidis A. Small-bowel capsule endoscopy and device-assisted enteroscopy for diagnosis and treatment of small-bowel disorders: European Society of Gastrointestinal Endoscopy (ESGE) Technical Review. Endoscopy. (2018) 50:423–46. doi: 10.1055/a-0576-0566, PMID: 29539652

[6] Fornaroli F Gaiani F Vincenzi F Bizzarri B Ghiselli A Kayali S. Applications of wireless capsule endoscopy in pediatric age: an update. Acta Biomed. (2018) 89:40–6. doi: 10.23750/abm.v89i9-S.7957, PMID: 30561394 PMC6502199

[7] Nuutinen H Kolho KL Salminen P Rintala R Koskenpato J Koivusalo A. Capsule endoscopy in pediatric patients: technique and results in our first 100 consecutive children. Scand J Gastroenterol. (2011) 46:1138–43. doi: 10.3109/00365521.2011.584900, PMID: 21615227

[8] Iwama I Shimizu H Nambu R Okuhira T Kakuta F Tachibana N. Efficacy and safety of a capsule endoscope delivery device in children. Eur J Gastroenterol Hepatol. (2019) 31:1502–7. doi: 10.1097/MEG.0000000000001513 31464784

[9] Jensen MK Tipnis NA Bajorunaite R Sheth MK Sato TT Noel RJ. Capsule endoscopy performed across the pediatric age range: indications, incomplete studies, and utility in management of inflammatory bowel disease. Gastrointest Endosc. (2010) 72:95–102. doi: 10.1016/j.gie.2010.01.016 20472231

[10] al-Bawardy B Locke G Huprich JE Fletcher JG Fidler JL Barlow JM. Retained capsule endoscopy in a large tertiary care academic practice and radiologic predictors of retention. Inflamm Bowel Dis. (2015) 21:2158–64. doi: 10.1097/MIB.0000000000000482, PMID: 26284295

[11] Atay O Mahajan L Kay M Mohr F Kaplan B Wyllie R. Risk of capsule endoscope retention in pediatric patients: a large single-center experience and review of the literature. J Pediatr Gastroenterol Nutr. (2009) 49:196–201. doi: 10.1097/MPG.0b013e3181926b01, PMID: 19561547

[12] Cohen SA. The potential applications of capsule endoscopy in pediatric patients compared with adult patients. Gastroenterol Hepatol. (2013) 36:92–6. doi: 10.1016/j.gastrohep.2012.10.007 23983653 PMC3754776

[13] Burgess CJ McIntyre EC Withers GD Ee LC. Comparing swallowing of capsule to endoscopic placement of capsule endoscopy in children. JGH Open. (2017) 1:11–4. doi: 10.1002/jgh3.12001, PMID: 30483526 PMC6207005

[14] Juan L-S Cernadas G Olivera PA Moore R. Prolonged intestinal transit time and its relation with capsule endoscopy diagnostic yield. Turk J Gastroenterol. (2022) 33:520–4. doi: 10.5152/tjg.2022.21251 35786621 PMC9317723

[15] Wu J Huang Z-H Wang Y-H Tang Z Lai L Xue A. Clinical features of capsule endoscopy in 825 children: a single-center, retrospective cohort study. Medicine. (2020) 99:e22864. doi: 10.1097/MD.0000000000022864, PMID: 33120825 PMC7581167

[16] Juanmartiñena Fernández JF Fernández-Urien Sainz I Zabalza Ollo B Saldaña Dueñas C Montañés Guimera M Elosua González A. Gastroduodenal lesions detected during small bowel capsule endoscopy: incidence, diagnostic and therapeutic impact. Rev Esp Enferm Dig. (2018) 110:102–8. doi: 10.17235/reed.2017.5114/2017, PMID: 29152990

